# Bacterial Nanocellulose as a Functional Ingredient for Texture Modification and Physicochemical Improvement of Goat Cheese During Refrigerated Storage

**DOI:** 10.3390/molecules31173049

**Published:** 2026-08-30

**Authors:** Hasbleidy Palacios-Hinestroza, María Camila López-Jaramillo, Julián Paul Martínez-Galán, Carlos Molina-Ramírez, Diego Mauricio Sánchez-Osorno

**Affiliations:** 1 Department of Basic Sciences, Campus Tlajomulco, University of Guadalajara, Tlajomulco de Zúñiga 45641, Mexico; hasbleidy.palacios@academicos.udg.mx; 2 Grupo de Investigación e Innovación Ambiental (GIIAM), Institución Universitaria Pascual Bravo, Cl. 73, No 73a-226, Medellín 050034, Colombia; m.lopezja@pascualbravo.edu.co; 3 Laboratório Alimentación y Nutrición Humana (LANH), Escuela de Nutrición y Dietética, Universidad de Antioquia UdeA, Cl. 70, No 52–21, Medellín 050010, Colombia; julian.martinez@udea.edu.co; 4 Grupo de Investigación en Química y Bioprospección de Productos Naturales (QUIBIP), Universidad del Magdalena, Cl. 29H3 No 22–01, Santa Marta 470004, Colombia; cmolinar@unimagdalena.edu.co; 5 Grupo de Investigación en Calidad y Productividad (QUALIPRO), Institución Universitaria Pascual Bravo, Cl. 73, No 73a-226, Medellín 050034, Colombia

**Keywords:** bacterial nanocellulose, goat cheese, texture, water activity, microstructure, functional ingredient

## Abstract

Bacterial nanocellulose (BNC) is a natural hydrocolloid with a high water-holding capacity and structuring potential in food systems. This study evaluated the effects of BNC addition at two concentrations (0.4% and 0.8%) on the physicochemical properties, texture profile, and microstructure of goat cheese during 21 days of refrigerated storage. Cheeses were characterized in terms of water activity (Aw), moisture content, and color (L*, a*, b*) and analyzed using texture profile analysis (TPA), Fourier-transform infrared spectroscopy (FTIR), and microscopy (optical, Cryo-SEM, and SEM). The addition of BNC significantly reduced water activity in a concentration-dependent manner; the 0.8% treatment reached the lowest Aw values by day 21. Moisture retention was improved, particularly at 0.4% BNC. Texture analysis showed that BNC reduced hardness and gumminess while increasing cohesiveness over time. FTIR spectra confirmed the incorporation of BNC into the cheese matrix, as evidenced by increased intensity in the C–H stretching region. Microstructural analysis demonstrated effective integration of nanocellulose fibers into the protein network, although higher concentrations led to localized fat agglomeration. These results indicate that BNC can serve as a clean-label ingredient to enhance the texture and stability of goat cheese, with potential applications in the development of fresh or reduced-fat style cheeses. Further sensory evaluation and compositional analysis (including fat content evaluation) are recommended to fully establish its practical utility.

## 1. Introduction

Goat cheese is an important product in several countries, particularly in Latin America. It is valued for its high nutritional value, distinctive flavor, and good digestibility. Despite these advantages, the production of high-quality goat cheese—especially fresh and reduced-fat varieties—continues to face several technological challenges. These products are often characterized by excessive moisture loss, a weak protein matrix, and pronounced syneresis, which can negatively affect cheese yield, shorten shelf life, and reduce consumer acceptance [1]. These problems have driven researchers and cheese manufacturers to investigate natural ingredients capable of reinforcing the cheese structure and enhancing its functional properties without relying on synthetic additives.

Bacterial nanocellulose (BNC) is considered a promising candidate for this purpose. BNC is a natural biopolymer produced by certain acetic acid bacteria, such as *Komagataeibacter medellinensis*. Unlike plant-derived cellulose, BNC is exceptionally pure, free of lignin and hemicellulose, and is characterized by high crystallinity, remarkable mechanical strength, and outstanding water-holding capacity (it can retain up to 100 times its own weight in water). In addition, BNC has a unique three-dimensional nanofibrillar network that allows it to effectively interact with both water molecules and proteins within food matrices [2]. Over the past decade, BNC has attracted increasing interest in the food industry as a multifunctional ingredient capable of serving as a fat replacer, thickening agent, stabilizer, and texture modifier. Several studies have demonstrated its potential in dairy applications, where it can reduce syneresis, enhance creaminess, and improve the mouthfeel of low-fat products [3,4,5,6].

When BNC is incorporated into cheese, its large specific surface area and abundant hydroxyl groups promote the formation of hydrogen bonds with water and casein. This interaction can modify the protein network, influence the water distribution, and contribute to a more stable and cohesive structure [7]. Moreover, BNC is biodegradable, biocompatible, and generally recognized as safe for food applications, aligning well with current trends toward sustainable and clean-label products [8].

Most studies conducted to date have focused on cheeses made from cow’s milk or other dairy products. Compared with cow’s milk, goat’s milk contains smaller fat globules and a distinct casein micelle assembly structure, which alters the water–protein–lipid interactions within the cheese matrix [9,10]. Therefore, the stability and textural modification efficacy of BNC cannot be directly inferred from studies on cow-milk cheese, highlighting the necessity of the present work. Relatively little attention has been paid to the specific performance of BNC in goat cheese, particularly with respect to its long-term physicochemical stability, texture development, and microstructural changes during refrigerated storage.

The concentrations of 0.4% and 0.8% (*v*/*w*) were selected based on previous reports of bacterial cellulose used as a fat mimetic or structuring agent in dairy systems (typically in the range of 0.3–1.0%) and on preliminary laboratory trials indicating that levels above approximately 1% markedly increased the viscosity of the milk and hindered proper curd formation. These two levels therefore represent a low and a moderate practical dose that still allow for conventional cheese manufacture [2,4].

The present study aimed to evaluate the effects of incorporating bacterial nanocellulose at two concentrations (0.4% and 0.8%) on the key quality attributes of goat cheese. Over a 21-day refrigerated storage period, the water activity, moisture content, color parameters, texture profile, FTIR spectra, and microstructural characteristics were evaluated using optical microscopy, cryogenic scanning electron microscopy (Cryo-SEM), and conventional scanning electron microscopy (SEM).

## 2. Results and Discussion

### 2.1. Dry Matter, Moisture, Color of Cheese, and Water Activity

All treatments showed a progressive decrease in water activity (Aw) throughout the storage period (Table 1 and Table 2). Two-way ANOVA indicated highly significant effects of treatment (*p* < 0.001), storage time (*p* < 0.001), and their interaction (*p* = 0.002) (Table 3). On day 6, Aw was significantly higher in cheeses containing BNC than in the control. By day 21, the 0.8% BNC treatment (BNC2) produced the lowest Aw value (0.839 ± 0.017), which was statistically different from both the 0.4% treatment and the control according to Tukey’s HSD test. This reduction indicates that BNC acts as an effective water-binding agent, immobilizing free water within the cheese matrix and thereby lowering water activity while helping to maintain structural integrity [3,5,11].

Moisture content also declined over time in all samples; however, the pattern differed among treatments (Table 3). Cheeses supplemented with BNC generally retained higher moisture levels than the control throughout storage, with the 0.4% treatment (BNC1) showing the highest retention (68.945% on day 6 versus 30.865% in the control). These differences were statistically significant and displayed a strong treatment × time interaction (*p* < 0.001). The large difference in moisture content can be attributed to the high water-holding capacity of BNC, which arises from its three-dimensional nanofibrillar network and the abundance of hydroxyl groups capable of forming hydrogen bonds with water and casein molecules. This physical entrapment and molecular interaction limit the release of free water during storage, even though adding salt during manufacture promotes syneresis [3,4,11,12,13].

Regarding color, the lightness parameter (L*) decreased significantly during the first 13 days of storage in all treatments (*p* < 0.001), indicating progressive darkening (Table 2). At day 6, the control cheese showed significantly higher L* values (73.10 ± 2.65) than the BNC-supplemented samples. The naturally whiter color of goat cheese is attributed to the very low concentration of β-carotene and other carotenoids in goat milk [14,15]. The slight reduction in L* observed in the BNC treatments may be related to changes in light scattering caused by the nanofibrillar structure embedded in the protein matrix; similar effects of nanofibrillar additives on the optical properties of dairy systems have been reported previously [5,16]. The a* parameter remained negative throughout storage, reflecting the characteristic slight greenish tint of fresh goat cheeses, while b* (yellowness) showed only minor variations.

Overall, the addition of BNC, particularly at 0.8%, was effective in reducing water activity while preserving acceptable moisture retention. These modifications are expected to contribute positively to the microbial stability and textural properties of goat cheese during refrigerated storage. The strong water-binding capacity of BNC, attributable to its high crystallinity and nanofibrillar architecture, enables it to function as a natural hydrocolloid that interacts with the casein network [5,7,17].

### 2.2. Morphology Analysis

The microstructure of the experimental goat cheeses was examined using optical microscopy and scanning electron microscopy (SEM and Cryo-SEM) to evaluate the distribution and interactions of fat globules, protein micelles, and bacterial nanocellulose within the cheese matrix.

#### 2.2.1. Optical Microscopy

**Figure 1:**
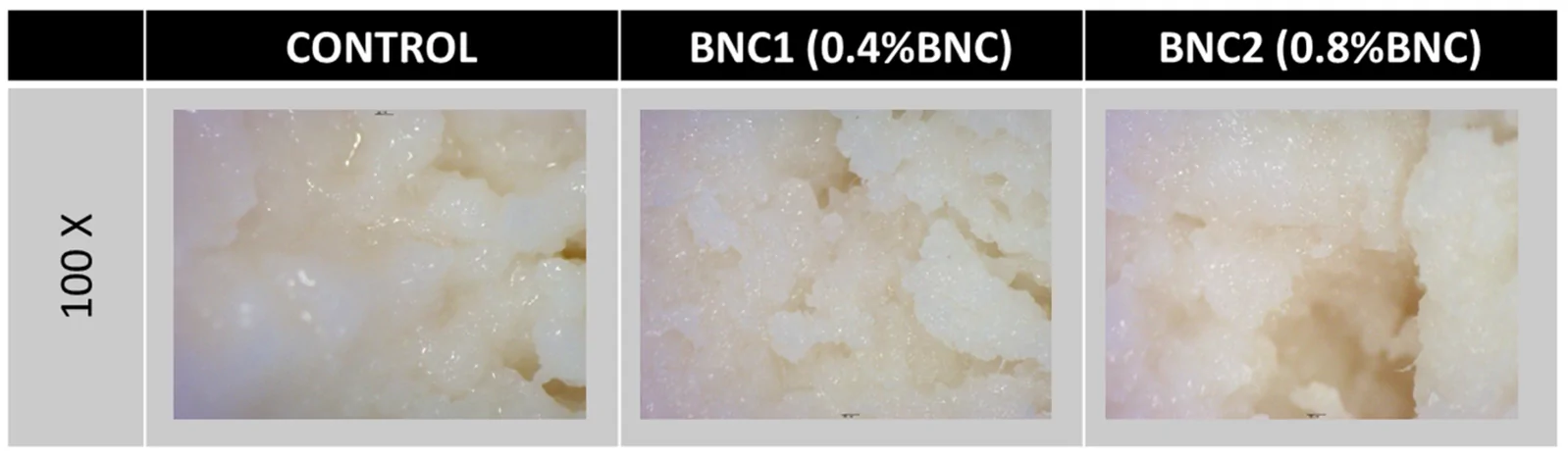
Optical microscopy of experimental goat cheese samples.

Optical micrographs of the samples are presented in Figure 1. The presence of fibrous structures was observed in samples with bacterial nanocellulose (BNC1 and BNC2). These fibers could be due to the added bacterial nanocellulose. The BNC was well distributed throughout the protein matrix, possibly due to a good compatibility between the nanocellulose and the cheese components. In comparison with the control, which showed a more homogeneous and less fibrous appearance, the BNC-containing samples exhibited a visibly more open matrix with embedded fibrous elements. Similar observations have been reported when bacterial cellulose was incorporated into dairy matrices [3,5,11].

#### 2.2.2. Cryo-SEM Analysis

**Figure 2:**
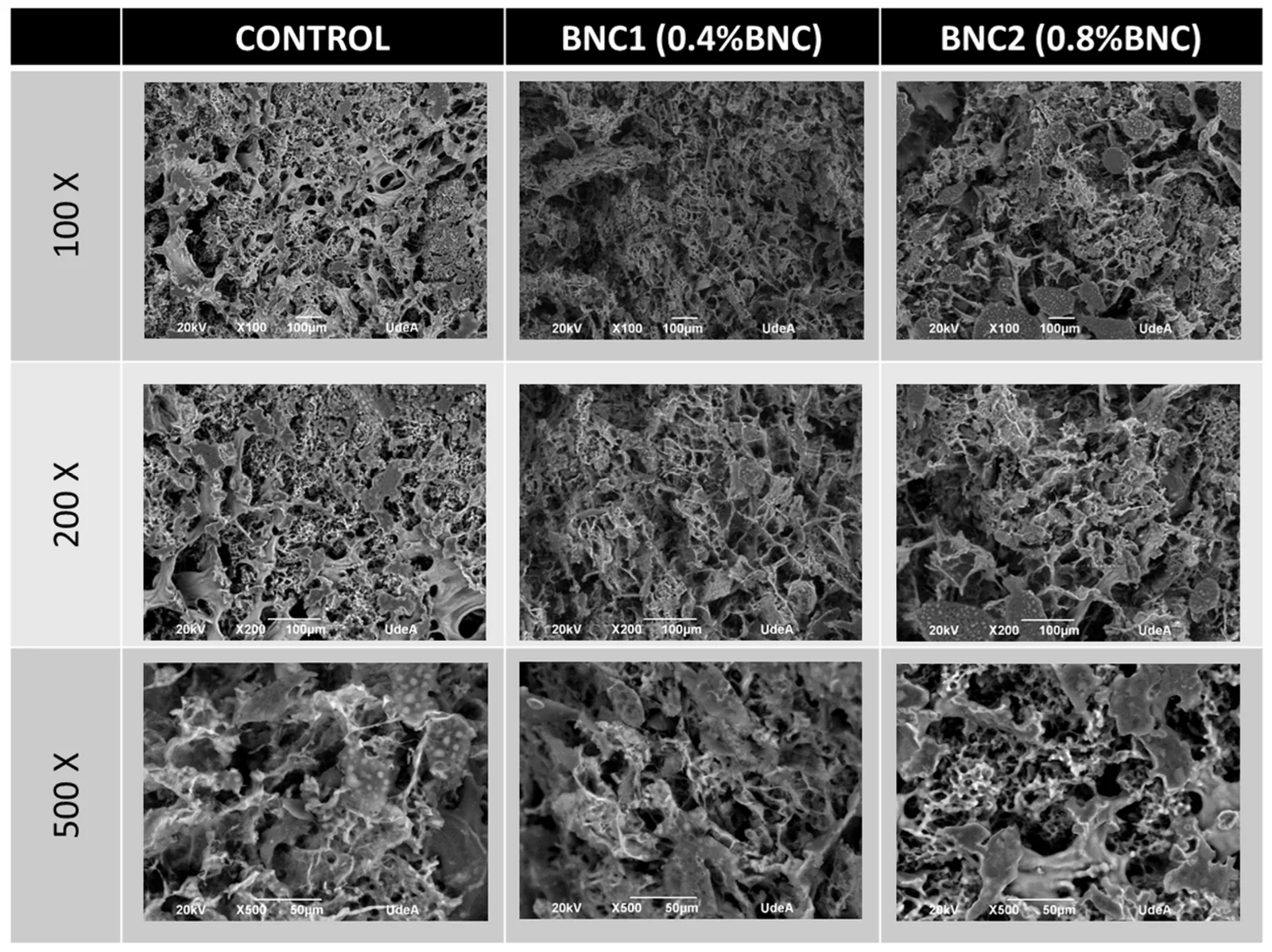
SEM CRYO micrographs of experimental goat cheese samples.

Micrographs of the goat cheese samples are shown in Figure 2. Both the control and the BNC1 treatment (0.4% BNC) samples exhibited a relatively homogeneous microstructure. In contrast, the BNC2 sample (0.8% BNC) displayed localized regions of apparent fat agglomeration. This observation suggests that higher concentrations of BNC may hinder the uniform dispersion of fat globules during cheese manufacture, possibly because the denser nanofibrillar network restricts free movement of the lipid phase. Previous studies have shown that structuring agents such as bacterial cellulose can modify fat globule distribution and interactions within the protein matrix of cheese [3,7,11,17].

#### 2.2.3. SEM Analysis

**Figure 3:**
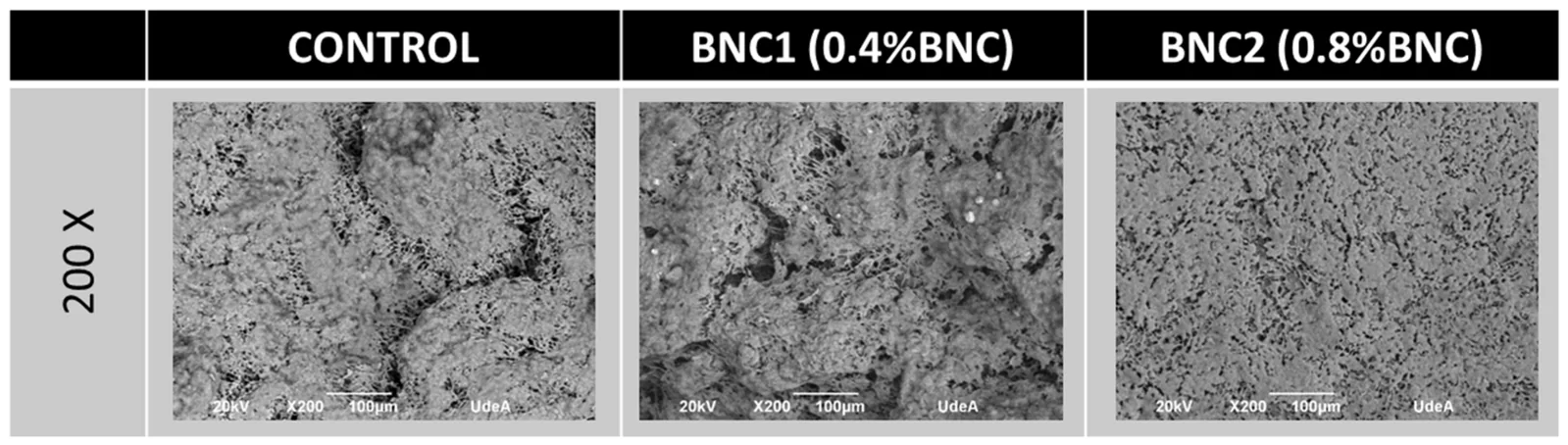
SEM micrographs of experimental goat cheese samples.

Conventional SEM micrographs (Figure 3) revealed clear differences in microstructure among treatments, particularly regarding porosity and the integration of bacterial nanocellulose. In the supplemented samples, the bacterial cellulose fibers formed an interconnected network within the protein matrix. This network was well integrated into the cheese structure, indicating good compatibility and acceptance of the nanocellulose by the goat cheese matrix. Similar network formation and structural reinforcement have been described when bacterial cellulose was added to food systems, where it acted as a natural hydrocolloid capable of modifying the microstructure and water distribution [5,11,18].

The control cheese presented a denser protein network with lower visible porosity, whereas cheeses containing BNC displayed a more open microstructure. These morphological differences are consistent with the texture profile results reported below, in which BNC-supplemented cheeses showed significantly lower hardness and gumminess than the control.

At 0.4% BNC, the nanocellulose integrated homogeneously without major disruption of the overall structure. At 0.8% BNC, although good integration was still observed, localized fat agglomeration and increased porosity were detected.

Bacterial nanocellulose is a water-binding and structuring agent that modified the protein–fat–water interactions in the cheese samples. These findings are consistent with previous studies that demonstrated that bacterial cellulose can be used as a functional ingredient for improving the texture and stability of dairy products [3,5,7].

These microstructural features help explain the reduction in hardness and the later increase in cohesiveness observed during storage: the open, fiber-reinforced network initially softens the matrix while progressive reorganization and local fat clustering at the higher dose contribute to subsequent firming.

### 2.3. Texture Profile Analysis (TPA)

The incorporation of BNC significantly modified the texture profile parameters of the goat cheese during storage (Table 4 and Table 5). Hardness was highest in the control sample throughout the 21-day storage period (3912–4341 g) and showed a slight upward trend over time. In contrast, the addition of BNC led to a concentration-dependent reduction in hardness. The BNC2 treatment (0.8% BNC) reached its lowest hardness on day 6 (471.5 g) before increasing sharply on day 13 (2434 g) and then stabilizing, while BNC1 (0.4% BNC) maintained intermediate and relatively stable values. This behavior indicates that BNC acts as a structural modifier, initially disrupting the dense casein matrix in a manner analogous to the polysaccharide-based fat replacers used in low-fat cheese systems [2,3,19]. The subsequent increase in hardness observed only in BNC2 after day 6 can be attributed to the progressive reorganization of the protein–BNC network and the formation of a more consolidated composite structure through self-assembly of cellulose nanofibrils [20]. This interpretation is consistent with the Cryo-SEM observation of localized fat agglomeration at the higher BNC concentration, which may also contribute to matrix firming during storage.

Cohesiveness increased in all samples over time. The most pronounced rise occurred in BNC2, which started at 0.162 on day 6 and reached 0.446 by day 21, eventually surpassing the control. This improvement suggests that BNC strengthens the internal binding capacity of the cheese matrix. Owing to its high water-holding capacity and large surface area, BNC retains moisture within the protein network, reducing syneresis and promoting a more cohesive structure [4].

Elasticity (springiness) was also influenced by BNC content. The control consistently exhibited the highest values. In BNC2, elasticity was initially low on day 6 but increased substantially by day 13, whereas in BNC1, it remained low and was more stable. The early reduction indicates that the nanofibrillar network interferes with the immediate elastic recovery of the casein gel; subsequent recovery during storage points to structural rearrangements and progressive protein–BNC interactions [2].

Gumminess followed a similar trend to hardness and remained significantly higher in the control. BNC2 showed a sharp increase between days 6 and 13 before stabilizing at intermediate values, while BNC1 maintained the lowest gumminess throughout. These results indicate that BNC can reduce the rubbery texture often associated with certain cheese varieties, which may be advantageous for the development of fresher or more spreadable products [21]. Adhesiveness values were generally more negative (higher stickiness) in the BNC-supplemented cheeses, particularly BNC1, consistent with the behavior of hydrocolloid-enriched dairy matrices.

The texture changes are consistent with the water-activity and FTIR results. The lower Aw recorded in BNC2, especially on day 21, supports the idea that BNC promotes the retention of water in a bound state, contributing to a more cohesive matrix without excessive hardness. The intensification of the C–H stretching band around 2900 cm^−1^ in the FTIR spectra further confirms the integration of BNC into the cheese structure and its influence on protein–water interactions.

### 2.4. Attenuated Total Reflection Fourier-Transform Infrared Spectroscopy (ATR-FT-IR)

**Figure 4:**
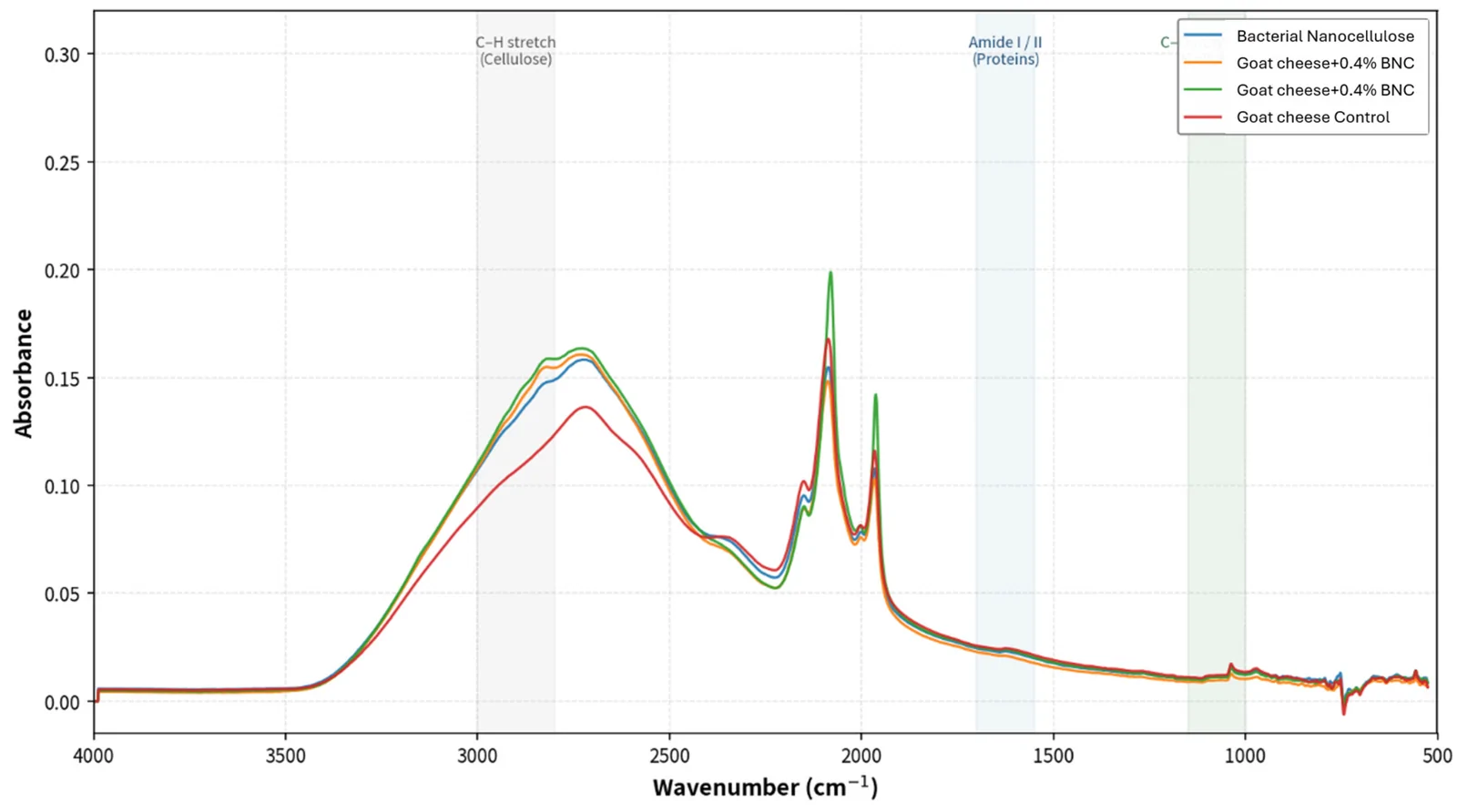
FTIR spectra of goat cheese samples and pure bacterial cellulose.

The FTIR spectra revealed clear differences between pure bacterial cellulose and the goat cheese matrix, as well as BNC concentration-dependent changes (Figure 4).

Pure bacterial cellulose display the characteristic absorption bands of cellulose I, including a broad O–H stretching band around 3340 cm^−1^, C–H stretching at ~2900 cm^−1^, and strong C–O stretching vibrations in the 1050–1030 cm^−1^ region, along with the diagnostic β-glycosidic linkage band near 890 cm^−1^ (Table 6). These characteristics show the high purity and crystalline nature of the bacterial nanocellulose used.

The control goat cheese displayed the typical dairy profile, with prominent Amide I (~1650 cm^−1^) and Amide II (~1540 cm^−1^) bands arising from casein and whey proteins, together with C–H stretching vibrations from the milk fat 2920–2850 cm^−1^ region.

In the BNC-supplemented cheeses, the intensity of the C–H stretching region near 2900 cm^−1^ increased with increasing BNC concentration. This enhancement is consistent with the additional CH_2_ groups contributed by bacterial cellulose. While the increase is primarily attributable to the superposition of BNC signals, the absence of major shifts in the positions of the Amide I and II bands suggests that the secondary structure of the proteins was not substantially disrupted. A moderate increase was also observed in the 1050–1030 cm^−1^ region. Although this range overlaps with carbohydrate signals from lactose, the progressive rise in absorbance that parallels the BNC dose, together with the concurrent increase in the C–H region, supports a contribution from the C–O stretching vibrations of the added nanocellulose [5,22].

Overall, the spectra indicate that BNC was successfully integrated into the goat cheese matrix without major alteration of the protein secondary structure, supporting its role as a structuring and water-binding agent.

**Table 6:** Characteristic FTIR absorption bands of bacterial cellulose and goat cheese samples.

Wavenumber (cm^−1^)	Assignment	Component	Reference(s)
3340–3300	O–H stretching (H-bonded)	Bacterial Cellulose	[3,5]
2920–2850	C–H stretching (CH_2_)	Bacterial Cellulose / Lipids	[5,12,23]
1740–1735	C = O stretching (ester carbonyl)	Milk fat (Triglycerides)	
1650–1635	Amide I (C = O stretching)	Cheese proteins (Casein)	[12,22]
1540–1530	Amide II (N–H bending + C–N)	Cheese proteins	
1430–1420	CH_2_ bending (crystalline)	Bacterial Cellulose	[5]
1165–1160	C–O–C asymmetric stretching	Bacterial Cellulose (glycosidic)	[5]
1055–1030	C–O stretching	Bacterial Cellulose / Lactose	[5,22]
900–890	C–H rocking (β-glycosidic)	Bacterial Cellulose (Cellulose I)	[5]

Notes: Band assignments are based on standard FTIR literature for bacterial cellulose and dairy products. Overlapping regions (e.g., 1050–1030 cm^−1^) may contain contributions from both nanocellulose and lactose.

## 3. Experimental Section

### 3.1. Production of Bacterial Nanocellulose

Bacterial nanocellulose (BNC) was produced by fermentation using *Komagataeibacter medellinensis* according to the method of Sánchez et al. (2024) [24]. The fermentation media were formulated from agro-industrial fruit residues (mainly mango and pineapple pomace). The fruit mixture was blended with water at a ratio of 1:2 (*w*/*v*). Fermentation was initiated by inoculating each medium with 10% (*v*/*v*) of the bacterial culture and was conducted under static conditions (no stirring or forced aeration) at 28 °C for 60 days. The initial pH of the medium was approximately 4.5–5.0. At the end of the fermentation period, the BNC pellicles that had formed at the air–liquid interface were collected, rinsed thoroughly with distilled water, and purified by immersion in a 5 wt% KOH solution for 14 h. The purified pellicles were washed repeatedly with water until a neutral pH was achieved and then mechanically fibrillated using a Masuko MKCA6–2 grinder (Masuko Sangyo Co., Ltd., Saitama, Japan) to obtain cellulose nanoribbons.

### 3.2. Preparation of the Standard Goat Cheese

Goat’s milk was filtered with a 100 µm sieve, pasteurized (75 ± 0.5 °C, 15 s), and cooled down to 38 ± 0.5 °C, followed by the addition of 5 mL/L of a 10% wt calcium chloride solution and 0.25 mL/L of rennet (strength: 1:10,000; Industrias Marshall, S.A. Medellín, Colombia), followed by gentle stirring. After a coagulation time of approximately 45 min, the curd was cut into 1 cm^3^ cubes, followed by heating (35 ± 0.5 °C), the addition of 2 g of sodium chloride, and gentle stirring for 10 min; then, about 80% of the whey was drained. Salting was carried out by adding 13 g/L milk and allowing the mixture to stand for 10 min. The curd was transferred to 300 g cylindrical molds (3 cm radius and 4 cm height) and kept at room temperature (20 ± 2 °C) for 2 h. Afterward, the molds were turned and left to stand for 1 h. Once the curd had drained, the molds were held in a cooling chamber at 4 ± 1 °C for 24 h. Finally, the cheeses were removed from the molds, wrapped in polyethylene film, and stored at 4 ± 1 °C for 6, 13, and 21 days before characterization. Production of each cheese variation was carried out in triplicate in a completely randomized design.

### 3.3. Preparation of Goat Cheese with Cellulose

Two different concentrations of bacterial nanocellulose in goat’s milk were tested, corresponding to 0.4% *v*/*w* (BNC1) and 0.8% *v*/*w* (BNC2). These solutions (bacterial nanocellulose in goat’s milk) were used instead of pure goat’s milk to prepare the cheese samples. The preparation process for these cheeses was the same as described above.

### 3.4. Dry Matter and Moisture

The dry matter and moisture content of the cheese samples were determined using a moisture balance (Shimadzu MOC 63u, Nakagyo-ku, Japan).

### 3.5. Determination of the Color of the Cheese Samples

The color of the cheese samples with different treatments was measured using an X-Rite SP62 colorimeter (X-Rite Inc., Grandville, MI, USA) and expressed as L* (lightness), a* (redness/greenness), and b* (yellowness/blueness) values.

### 3.6. Water Activity

A Rotronic HygroPalm HP23-AW-A meter (Rotronic, Bassersdorf, Switzerland) was employed to measure the activity water (aw) of the cheese samples; according to aw values, the cheeses were classified as soft cheeses (ripened for 30 days) (aw: 0.97–0.99) (18/84), semi-hard cheeses (ripened for 30 to 60 days) (aw: 0.96–0.93) (41/84) or hard cheeses (ripened for more than 60 days) (aw: 0.92–0.79) (25/84).

### 3.7. FT-IR

Cheese samples were analyzed by ATR-FTIR using a Nicolet 6700 spectrophotometer (Thermo Fisher Scientific, Waltham, MA, USA) in the 4000–400 cm^−1^ range using a diamond crystal. Spectra were recorded at a resolution of 4 cm^−1^ with an accumulation of 64 scans.

### 3.8. Rheological Measurements

Cheese rheological properties were determined using the uniaxial compression method. Cylindrical samples (r ¼ 5 mm; h ¼ 10 mm) were cut from the cheeses at 4 °C. Samples were equilibrated at 37 °C for 1 h and compressed 2 times to 50% of their original height at a rate of 0.4 mm s^−1^ on a TA-XT2 model texturometer (Mono Research Laboratories, Brampton, ON, Canada). Ten replicate samples were analyzed for each cheese. Young’s modulus was calculated from the initial slope of the stress-deformation profile during the first compression, and texture profile analysis parameters (fracturability, elasticity and cohesiveness) were calculated according to the method of Lamothe et al. (2012) [25].

### 3.9. Optical Microscopy

The morphology and microstructure of the cheese samples were characterized via optical microscopy using an Olympus BX51 optical microscope (Olympus Corporation, Tokyo, Japan), following the approach described by Auty (2018) [26]. Representative micrographs were analyzed and scaled to a resolution of 100 µm.

### 3.10. Cryogenic Scanning Electron Microscopy (Cryo-SEM)

The microstructure of the goat cheese samples was characterized by Cryo-SEM using a JEOL JSM 6490 LV microscope (Tokyo, Japan) equipped with a PP3000T Cryo Transfer System (Quorum Technologies, East Sussex, UK). Small cheese pieces were rapidly frozen in slush nitrogen (−210 °C), fractured at −150 °C, and etched for 5–10 min at −90 °C. The fractured surfaces were sputter-coated with platinum under cryogenic conditions and observed at an accelerating voltage of 15–20 kV, with the sample stage maintained at −150 °C. Micrographs were obtained at magnifications ranging from 1000× to 5000×. Image analysis was performed using ImageJ software version 1.54h to evaluate microstructural features at a 100 µm scale.

### 3.11. SEM

The morphology and the microstructure of the cheese samples were characterized by scanning electron microscopy (SEM) using a JEOL JSM 6490 LV under high vacuum and operating at an acceleration voltage of 20 kV. The samples were sputtered-coated with gold before observation. Micrographs were obtained at a magnification of 4000 and compared both visually and using Image J software at a resolution of 100 µm.

### 3.12. Statistical Analysis

All analyses of cheeses were repeated three times. Data are expressed as means and standard deviations. Differences with *p* < 0.05 were considered statistically significant.

All experiments were conducted in triplicate using a completely randomized design (CRD). Data were expressed as the mean ± standard deviation (SD). To evaluate the effects of the treatment factor (BNC concentration: control, 0.4%, and 0.8%), storage time (6, 13, and 21 days), and their interaction (treatment × storage time), a two-way analysis of variance (ANOVA) was performed for physicochemical parameters (water activity Aw, moisture content, and L*, a*, b* color coordinates) and texture profile analysis (TPA) parameters (cohesiveness, elasticity, hardness, and gumminess). Homogeneity of variance and normality of residuals were verified prior to testing. When significant differences were detected (*p* < 0.05), Tukey’s Honestly Significant Difference (HSD) post hoc test was applied for multiple comparisons of means. Additionally, the magnitude of the observed effects was determined using partial eta squared, where values greater than 0.14 were interpreted as large effect sizes. Statistical processing was executed using SPSS v.26.0.

## 4. Conclusions

The incorporation of bacterial nanocellulose into goat cheese significantly affected its physicochemical and textural properties during refrigerated storage. The 0.8% concentration was particularly effective in lowering water activity while producing a softer and more cohesive cheese. At 0.4%, the main practical advantage was improved moisture retention. The texture profile analysis indicated that BNC functioned as a structural modifier, reducing hardness and gumminess while preserving overall structural integrity. The microstructural observations confirmed good integration of the nanocellulose fibers into the protein matrix, although some fat agglomeration occurred at the higher concentration. The FTIR results further supported the successful incorporation of BNC into the cheese structure.

Taken together, the findings suggest that bacterial nanocellulose has potential as a clean-label ingredient for improving the texture and stability of goat cheese, especially in the development of fresher or reduced-fat style products. Nevertheless, sensory evaluation and determination of fat content will be necessary to confirm consumer acceptance and to optimize the concentration according to the specific type of cheese being targeted. Future studies should also explore the long-term microbial stability of BNC-supplemented cheeses and the precise molecular interactions between BNC and the casein network.

## Figures and Tables

**Table 1:** Characteristics of goat cheese samples.

Sample		Aw		Humidity	Color		
		Aw	T (°C)	%H	L	a	b
BNC1	6	0.958	20.600	68.945	66.678	−0.545	8.748
BNC1	13	0.915	19.850	57.535	48.673	−0.618	7.703
BNC1	21	0.892	19.700	60.043	51.488	−0.735	8.270
BNC2	6	0.942	22.900	56.343	64.060	−0.825	8.928
BNC2	13	0.881	22.125	43.045	48.095	−0.670	8.945
BNC2	21	0.839	21.475	45.575	46.438	−0.803	8.890
Control	6	0.914	23.975	30.865	73.103	−1.090	10.288
Control	13	0.904	22.575	34.678	51.733	−0.830	8.783
Control	21	0.871	22.525	33.775	54.830	−1.023	9.018

**Table 2:** Mean ± standard deviation of the physicochemical parameters of goat cheese samples.

Treatment	Day 6	Day 13	Day 21
Aw			
Control	0.914 ± 0.009 b	0.904 ± 0.011 b	0.871 ± 0.009 b
BNC1 (0.4%)	0.958 ± 0.023 a	0.915 ± 0.004 a	0.892 ± 0.024 a
BNC2 (0.8%)	0.942 ± 0.009 a	0.881 ± 0.005 b	0.839 ± 0.017 b
Humidity (%)			
Control	30.865 ± 2.968 c	34.677 ± 3.906 c	33.775 ± 3.915 c
BNC1 (0.4%)	68.945 ± 2.247 a	57.535 ± 1.453 a	60.042 ± 2.803 a
BNC2 (0.8%)	56.998 ± 1.337 b	43.045 ± 1.506 b	45.575 ± 1.698 b
L*			
Control	73.102 ± 2.650 b	51.733 ± 3.982 a	54.830 ± 4.829 a
BNC1 (0.4%)	66.677 ± 2.615 a	48.672 ± 6.679 a	51.487 ± 2.265 a
BNC2 (0.8%)	64.060 ± 1.383 a	48.095 ± 3.680 a	46.438 ± 1.138 a
a*			
Control	−1.090 ± 0.094 c	−0.830 ± 0.029 a	−1.022 ± 0.121 a
BNC1 (0.4%)	−0.545 ± 0.006 a	−0.618 ± 0.173 a	−0.735 ± 0.207 a
BNC2 (0.8%)	−0.825 ± 0.072 b	−0.670 ± 0.093 a	−0.803 ± 0.088 a
b*			
Control	10.287 ± 0.522 b	8.782 ± 1.069 a	9.018 ± 1.366 a
BNC 1 (0.4%)	8.747 ± 0.392 a	7.703 ± 0.795 a	8.270 ± 0.474 a
BNC2 (0.8%)	8.928 ± 0.442 a	8.945 ± 0.906 a	8.890 ± 0.853 a

Notes: BNC1 = goat cheese containing 0.4% bacterial nanocellulose; BNC2 = goat cheese containing 0.8% bacterial nanocellulose. Different letters (a, b, c) indicate statistically significant differences among means according to Tukey’s HSD post hoc test (*p* < 0.05).

**Table 3:** Two-way analysis of variance (ANOVA) of the evaluated physicochemical parameters.

Variable	Source of Variation	F	*p*-Value	Partial η^2^
Aw	Treatment	19.60	<0.001	0.162
	Storage time	76.74	<0.001	0.634
	Treatment × storage time	5.63	0.002	0.093
Humedad (%)	Treatment	372.29	<0.001	0.843
	Storage day	25.58	<0.001	0.058
	Treatment × storage time	15.13	<0.001	0.069
L* (color)	Treatment	11.32	0.0003	0.093
	Storage day	95.21	<0.001	0.782
	Treatment × storage time	0.86	0.498 (ns)	0.014

Notes: ns = not significant (*p* > 0.05). Partial eta squared (η^2^*p*) represents the proportion of variance explained by each source of variation. Values greater than 0.14 are considered large effect sizes.

**Table 4:** Texture profile of the goat cheeses.

Sample		Cohesiveness	Adhesiveness, _1_ (g.s)	Adhesiveness, _2_ (g.s)	Elasticity	Hardness (g)	Gumminess
BNC1	6	0.309			0.185	1092.883	337.786
BNC1	13	0.368	−27.287	−24.443	0.494	930.260	339.405
BNC1	21	0.375	−16.772	−13.511	0.493	1070.876	401.413
BNC2	6	0.162			0.113	471.492	76.621
BNC2	13	0.412			0.619	2434.221	1005.619
BNC2	21	0.446	−21.973	−20.812	0.605	2268.343	1011.697
CONTROL	6	0.336			0.475	3912.591	1323.221
CONTROL	13	0.428	−10.939	−13.298	0.943	3984.258	1702.477
CONTROL	21	0.487	−8.025	−2.488	0.905	4341.425	2115.424

**Table 5:** Statistical analysis of TPA parameters during refrigerated storage.

Parameter	Treatment	Day 6	Day 13	Day 21
**Cohesiveness**	Control	0.334 ± 0.006 b	0.404 ± 0.005 a	0.472 ± 0.031 c
	BNC1 (0.4%)	0.307 ± 0.017 a	0.376 ± 0.043 a	0.362 ± 0.012 a
	BNC2 (0.8%)	0.156 ± 0.041 b	0.398 ± 0.022 a	0.454 ± 0.023 b
**Elasticity** **(Springiness)**	Control	0.507 ± 0.057 b	0.937 ± 0.033 b	0.910 ± 0.026 c
	BNC1 (0.4%)	0.189 ± 0.026 a	0.457 ± 0.070 a	0.468 ± 0.035 a
	BNC2 (0.8%)	0.063 ± 0.064 a	0.590 ± 0.072 a	0.615 ± 0.063 b
**Hardness (g)**	Control	3870.567 ± 250.103 c	4009.067 ± 79.274 c	4403.367 ± 143.922 c
	BNC1 (0.4%)	1163.433 ± 122.707 a	827.667 ± 107.691 a	1033.700 ± 122.218 a
	BNC2 (0.8%)	395.300 ± 74.968 b	2501.667 ± 48.702 b	2235.667 ± 90.477 b
**Gumminess**	Control	1278.333 ± 112.440 c	1698.067 ± 100.875 c	2092.433 ± 39.274 c
	BNC1 (0.4%)	386.433 ± 80.315 a	248.733 ± 39.552 a	428.267 ± 122.982 a
	BNC2 (0.8%)	102.033 ± 54.987 b	1046.200 ± 61.860 b	1047.567 ± 115.923 b

Note: Values are expressed as mean ± standard deviation (n = 3 simulated replicates based on reported means). Different letters within the same column indicate significant differences according to Tukey’s HSD test (*p* < 0.05).

## Data Availability

The original contributions presented in this study are included in the article. Further inquiries can be directed to the corresponding author.
